# Clinical characteristics of yamakagashi (*Rhabdophis tigrinus*) bites: a national survey in Japan, 2000–2013

**DOI:** 10.1186/2052-0492-2-19

**Published:** 2014-03-06

**Authors:** Toru Hifumi, Atsushi Sakai, Akihiko Yamamoto, Masahiro Murakawa, Manabu Ato, Keigo Shibayama, Akihiko Ginnaga, Hiroshi Kato, Yuichi Koido, Junichi Inoue, Yuko Abe, Kenya Kawakita, Masanobu Hagiike, Yasuhiro Kuroda

**Affiliations:** Emergency Medical Center, Kagawa University Hospital, Toru Hifumi, 1750-1 Ikenobe, Miki, Kita, Kagawa, 761-0793 Japan; Japan Snake Institute, 3318 Yabutsuka, Ota, Gunma, 379-2301 Japan; Department of Bacteriology II, National Institute of Infectious Disease, Gakuen 4-7-1, Musashimurayama-shi, Tokyo, 208-0011 Japan; Department of Internal Medicine, Kaizuka Hospital, Hakosaki 7-7-27, Higashi-kuFukuoka, 812-0053 Japan; Department of Immunology, National Institute of Infectious Disease, Toyama 1-23-1, Shinjuku-ku, Tokyo, 162-8640 Japan; The Chemo-Sero-Therapeutic Research Institute (KAKETSUKEN), 1-6-1 Okubo, Kita-ku, Kumamoto-shi, Kumamoto, 860-8568 Japan; Division of Critical Care Medicine and Trauma, National Hospital Organization Disaster Medical Center, 3256 Midori-cho, Tachikawa, Tokyo, 190-0014 Japan; Division of Critical Care Medicine and Trauma, Yamanashi Prefectural Central Hospital, 1-1-1 Fujimi, Kofu, Yamanashi, 400-8506 Japan

**Keywords:** Yamakagashi, *Rhabdophis tigrinus*, Antivenom, Disseminated intravascular coagulation, Fibrinolytic phenotype

## Abstract

**Background:**

Yamakagashi (*Rhabdophis tigrinus)* is a species of pit viper present throughout Russia and Eastern Asia. Although *R. tigrinus* venom is known to induce life-threatening hemorrhagic symptoms, the clinical characteristics and effective treatment of *R. tigrinus* bites remain unknown. The present study aimed to clarify these issues.

**Methods:**

Records in the Japan Snake Institute between 2000 and 2013 were retrospectively investigated. The following were determined: patient characteristics, coagulation and fibrinolytic system abnormalities, effect of antivenom treatment, and outcomes.

**Results:**

Nine patients (all males; median age, 38 years) with *R. tigrinus* bites were identified. On admission, the median levels of fibrinogen and fibrinogen degradation products, and platelet counts were 50 mg/dL, 295 μg/mL, and 107,000/mm^3^, respectively. The median (minimum–maximum) disseminated intravascular coagulation (DIC) score defined by the Japanese Association of Acute Medicine was 8 (1–8). Antivenom was administered to seven patients, with a median interval of 35 h between bite and antivenom administration. All patients treated with antivenom survived, and the in-hospital mortality rate was 11%.

**Conclusions:**

Patients with *R. tigrinus* bites presented with DIC of a fibrinolytic phenotype, which can result in life-threatening injury unless appropriate antivenom and DIC treatment are provided.

## Background

Yamakagashi (*Rhabdophis tigrinus*) is a species of pit viper. It is present throughout Russia and Eastern Asia, including China, Taiwan, Korea, and Japan, but excluding Ryukyu Islands [1]. *R. tigrinus* venom is known to induce life-threatening hemorrhagic symptoms similar to those of the rattlesnake and other crotaline snakes [2–4]. However, the clinical characteristics and effective treatment of *R. tigrinus* bites remain unknown.

*R. tigrinus* antivenom was traditionally manufactured by the immunization of rabbits and goats. However, because of low supply, manufacture has more recently been based on immunizing horses and is supported by Health Science Grants (1998–1999) from the Ministry of Health, Labour and Welfare in 2000 [1]. In addition, the management of *R. tigrinus* bites in intensive care has progressed dramatically in this century [5].

This study aimed to elucidate the clinical characteristics of *R. tigrinus* bites and to clarify the effectiveness of antivenom treatment.

## Methods

The Japan Snake Institute records were retrospectively investigated for the period between January 1, 2000 and November 30, 2013. The study was approved by the institutional review board at the National Disaster Medical Center.

### Diagnosis of *R. tigrinus*bites

No definite diagnostic criteria exist. Diagnosis of *R. tigrinus* bites required lots of experience of clinical practice. Compared with Mamushi (*Gloydius blomhoffii*) bites, no apparent edema or pain is typically observed at the bite site [6]. However, *R. tigrinus* venom induces a fatal coagulopathy, which results in extensive hemorrhage [7]. Laboratory data usually show severe hypofibrinogenemia, which is considered both a sensitive and a specific diagnostic marker [6]. The disseminated intravascular coagulation (DIC) diagnostic criteria for critically ill patients were used, as outlined by the Japanese Association of Acute Medicine (JAAM criteria) [8]; DIC was defined as a total score of ≥4.

### Treatment of *R. tigrinus*bites

Fibrinogen levels <100 mg/dL are considered appropriate for antivenom administration in clinical practice. The antivenom used against *R. tigrinus* bites was experimentally manufactured and was effective against bites by snakes belonging to the genus *Rhabdophis*[1]. In total, 1,369 vials were produced and stored at two institutes: the Japan Snake Institute (Gunma) and Kaketsuken (Kumamoto).

Severe adverse effects refer to anaphylactic shock in which the patient was at risk of death because of antivenom administration.

In clinical practice, physicians managing patients with snake bites usually ask for the assistance of the Japan Snake Institute, where diagnosis is confirmed according to laboratory data and clinical symptoms. Antivenom is then dispatched to the treating physician by police car throughout Japan, other than in the Kyusyu region. In the Kyusyu region, Kaketsuken supplies antivenom to the physicians in the presence of the patient. Antivenom is supplied only by these two institutes. Clinical data was routinely collected, and all cases of *R. tigrinus* bites were recorded in the Japan Snake Institute.

### Data collection

The following parameters were recorded: age, gender, comorbidities, laboratory data, and DIC score, as well as treatment-related factors, including the adverse effects of antivenom, and the outcome at hospital discharge.

## Results

**Figure 1:**
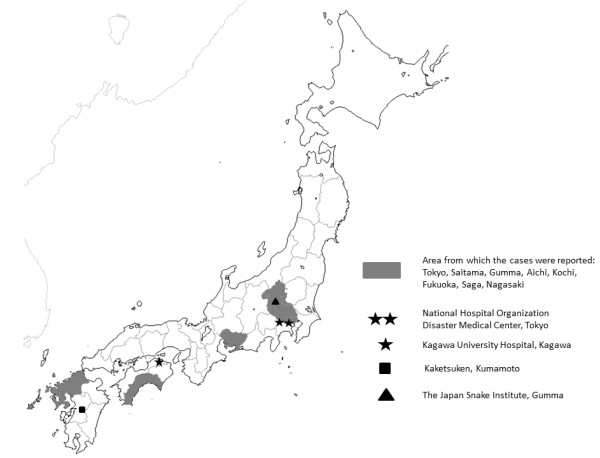
**A map showing the relative locations of cases of** ***Rhabdophis tigrinus*** **bites.** Tokyo, Saitama, Gunma, Aichi, Kochi, Fukuoka, Saga, and Nagasaki Prefectures.

Over the 14-year study period, nine patients were identified; the patient characteristics are summarized in Table 1. The area where *R. tigrinus* bites were reported was limited to four regions: Kanto, Chubu, Shikoku, and Kyusyu (Figure 1). All nine patients were male, with a median age of 38 years (5–81).

**Table 1:** **Characteristics of patients with** ***Rhabdophis tigrinus*** **bites and laboratory data on admission**

Case	Area	Age	Gender	Comorbidities	WBC (/mm ^3^ )	Plt (×10 ^4^ /mm ^3^ )	Fibrinogen (mg/dL)	PT-INR	FDP (μg/mL)	DIC score
1	Tokyo	54	M	LC	3,500	10.7	189	1.08	3.5	1
2	Fukuoka	75	M	DM,HT	17,300	1.8	50	2.62	271.5	8
3	Nagasaki	38	M	None	10,000	12.5	34.8	ODL	ODL	3
4	Saga	13	M	None	8,300	7.3	30	ODL	393	8
5	Fukuoka	40	M	None	9,840	19.8	50	8	592	5
6	Aichi	13	M	None	11,300	13.1	25	3.8	453	8
7	Gunma	37	M	None	10,100	3.9	30	2.81	295	8
8	Saitama	5	M	MR	25,800	22	50	7.46	209	5
9	Kochi	81	M	None	6,330	7.2	50	2.71	262	8
Summary		38 (5–81)	Male, 100%		10,000 (3,500–25,800)	10.7 (1.8–22)	50 (25–189)	3.8 (1.08–ODL)	295 (3.5–ODL)	8 (1–8)

Age, WBC, Plt, Fibrinogen, PT-INR, FDP, and DIC scores are expressed as median (minimum–maximum). WBC, white blood cell; Plt, platelet count; PT-INR, prothrombin time international ratio; FDP, fibrinogen degradation products; DIC, disseminated intravascular coagulation; ODL, over detection limit; LC, liver cirrhosis; DM, diabetes mellitus; HT, hypertension.

On admission, the median levels of fibrinogen and fibrinogen degradation products (FDP) and platelet counts were 50 mg/dL, 295 μg/mL, and 107,000/mm^3^, respectively. The median (minimum–maximum) DIC score was 8 (1–8) (Table 1). Antivenom was administered to seven patients, and the median interval between bite and antivenom administration was 35 h; no apparent adverse effects were observed. DIC was treated using clotting factor replacement with fresh frozen plasma (FFP) and protease inhibitors in three patients (Table 2). All patients treated with antivenom survived. However, the in-hospital mortality rate was 11% for all patients because a patient who did not receive antivenom died.

**Table 2:** **Treatment details and outcome**

Case	Gabexate mesilate/nafamostat mesilate	FFP	RCC	***R. tigrinus*** antivenom	Time interval between getting yamakagashi bites and antivenom administration (h)	Adverse effects related with antivenom	Severe adverse effects	ICU days	Hospital days	Outcome
1	No	No	No	No				2	2	Survival
2	No	Yes	Yes	No				6	6	Dead
3	No	No	No	Yes	5.5	No	No	2	4	Survival
4	Yes	No	No	Yes	24	No	No	0	6	Survival
5	No	No	No	Yes	28	No	No	0	6	Survival
6	No	No	No	Yes	35	No	No	0	7	Survival
7	No	No	No	Yes	35	No	No	2	4	Survival
8	Yes	Yes	Yes	Yes	60	No	No	0	14	Survival
9	Yes	Yes	No	Yes	60	No	No	0	6	Survival
Summary	Yes, 33%	Yes, 33%	Yes, 22%	Yes, 78%	35 (5.5–60)	Yes, 0%	Yes, 0%	0 (0–6)	6 (2–14)	Survival, 89%

Data are expressed as median (minimum–maximum) for the length of time from Yamakagashi bite to antivenom administration, ICU days, and hospital days. FFP, fresh frozen plasma; RCC, red cell concrete.

Case 1 (a 54-year-old man) with severe liver cirrhosis survived without administering antivenom. He had been bitten on his right hand by *R. tigrinus*. Initially, he demonstrated decreased platelet counts and fibrinogen levels without increased FDP levels. He was closely followed up, and both platelet counts and fibrinogen levels were not decreased on the second day of admission, which was considered to be caused by lower venom adsorption. Decreased platelet counts and fibrinogen levels on admission were caused by severe liver cirrhosis.

Case 2 (a 75-year-old man) died because of intracranial hemorrhage caused by DIC. He had been bitten on his left hand by a small snake and presented to the local clinic the next day with bleeding from the bite site. He was referred to the general hospital for further treatment and admitted without antivenom therapy. Two days after the bite, he developed disturbance of consciousness (Glasgow coma scale, 3) with 7-mm pupil dilation. Intracranial hemorrhage (bilateral acute epidural hemorrhage and subcortical hemorrhage) with cerebral herniation was revealed by computed tomography. Severe hypofibrinogenemia (50 mg/dL) also developed, and *R. tigrinus* was ultimately considered the definitive diagnosis. Antivenom was not administered at this late stage because of irreversible brain dysfunction. The patient died 8 days after the *R. tigrinus* bite.

## Discussion

In the present study, we demonstrated the clinical characteristics of *R. tigrinus* bites that were effectively treated with antivenom administration and provided an outline of an untreated case. Compared with the incidence of Mamushi bites estimated, which is approximately 1,000 cases annually [9], the incidence of *R. tigrinus* bites is extremely rare; however, there is a possibility that mild cases without severe coagulopathy like case 1 were not reported and that some physicians may have diagnosed *R. tigrinus* bites as nonvenomous [1]. Case 2 presented in this report may serve as a reminder of the risks of such an approach and of the need for a high index of suspicion.

*R. tigrinus* venom shows strong plasma coagulant activity, with prothrombin activating effects and weak thrombin-like effects [10]. Once *R. tigrinus* venom is absorbed into the circulation, it activates prothrombin and thereby excessive coagulation. Disseminated fibrin formation ensues and fibrinolysis is activated, resulting in hypofibrinogenemia and increased FDP levels [10, 11]. The current survey revealed that *R. tigrinus* bites caused severe DIC with a fibrinolytic phenotype. Based on the current data, it appears that following bites, *R. tigrinus* antivenom administration and appropriate DIC treatment can lead to complete recovery, even in the presence of severe DIC [12, 13].

Several days from *R. tigrinus* bites were required to administer antivenom in the present survey because of both the inconvenient supply of antivenom and the delays in diagnosis. Compared with the more common Mamushi bites, which are typically rapidly progressive, there appears to be a longer therapeutic window for administering *R. tigrinus* antivenom [14, 15]. DIC with the fibrinolytic phenotype is usually not accompanied with multiple organ dysfunction, and hemorrhage is the major concern. The excessive fibrino/fibrinogenolysis without fibrin formation and life-threatening bleeding are contributed by the expression of tissue plasminogen activator (t-PA) [16, 17]. Excess t-PA is secondarily induced by hypoperfusion in severe traumatic injury [16] and hypoxia [18, 19]. *R. tigrinus* may therefore have neither a t-PA effect in itself [6] nor induce excessive t-PA. Indeed, patients in the present study did not develop severe hypoperfusion at any stage during hospitalization, including the period before the administration of *R. tigrinus* antivenom*.* Therefore, antivenom was considered effective beyond the initial acute phase. Further study is required to evaluate the indications and proper timing of antivenom administration on the basis of the mechanism described.

Adverse reactions to antivenom were negligible. Although the numbers in this survey are too low to make an assessment, the adverse reaction rate may be lower than the 2.4%–9% rate observed with Mamushi antivenom [9, 20]. However, since both *R. tigrinus* and Mamushi antivenom are manufactured by immunizing horses, we should remain vigilant to the risk of adverse events such as anaphylaxis and serum sickness disease [21]. The great concern with current *R. tigrinus* bite treatment is that although the antivenom is effective, it is only experimentally manufactured by regional health laboratories [1]. Therefore, the sterility and safety are not guaranteed [1]. The Ministry of Health, Labour and Welfare of Japan has launched a research group to evaluate the safety and efficacy of antivenom and to organize and maintain information on *R. tigrinus* bites in 2013. Two hospitals have been selected as specialist centers for *R. tigrinus* bite treatment: The National Hospital Organization Disaster Medical Center, Tokyo and Kagawa University Hospital, Kagawa. Clinical characteristics and treatment details, including the adverse effects of antivenom, are recorded for all cases and analyzed to ensure proper safety. In addition, patients with *R. tigrinus* bites are covered by clinical research insurance to provide compensation in the event of adverse effects resulting from antivenom administration.

There are many limitations to this study. A major limitation is the fact that many cases remain undiagnosed or misdiagnosed because of the unfamiliar symptoms presented by this rare snakebite. In addition, we did not obtain specific clinical details, including coagulation markers, which would have been beneficial. Finally, as this was a retrospective analysis, the details of DIC were not obtained, such as the level of soluble fibrin and plasminogen activator inhibitor and treatment.

## Conclusions

*R. tigrinus* bites are rare in clinical practice but demonstrated DIC with the fibrinolytic phenotype, which can result in life-threatening injury unless appropriate antivenom and DIC treatment are provided. Throughout the at-risk Asian countries, critical care physicians should be aware of this injury and its treatment.
